# Artificial Intelligence in Materials Science and Engineering

**DOI:** 10.3390/ma19091808

**Published:** 2026-04-29

**Authors:** Piotr Lacki, Janina Adamus, Anna Derlatka, Wojciech Więckowski, Krzysztof Cpałka

**Affiliations:** 1 Department of Civil Engineering, Czestochowa University of Technology, Dabrowskiego 69, 42-201 Czestochowa, Poland; janina.adamus@pcz.pl (J.A.); anna.derlatka@pcz.pl (A.D.); 2 Department of Mechanical Engineering, Czestochowa University of Technology, Dabrowskiego 69, 42-201 Czestochowa, Poland; wojciech.wieckowski@pcz.pl; 3 Department of Artificial Intelligence, Czestochowa University of Technology, Dabrowskiego 69, 42-201 Czestochowa, Poland; krzysztof.cpalka@pcz.pl

The collection of 20 scientific articles published within the Special Issue titled “Artificial Intelligence in Materials Science and Engineering” provides a comprehensive overview of contemporary materials engineering, where traditional research methods are increasingly bolstered by advanced digital tools. These publications, featured in the ‘Materials Simulation and Design’ section, demonstrate how integrating Artificial Intelligence (AI) and numerical methods within Materials Science and Engineering accelerates the development of innovative technologies. This Special Issue serves as an interdisciplinary bridge connecting two often separate worlds: computer science theorists and materials engineering practitioners. AI researchers (computer scientists, mathematicians) frequently seek real-world, complex datasets to validate their models. Conversely, materials engineers possess unique experimental data but may lack the specialized expertise to implement techniques such as various metaheuristic optimization techniques exemplified by the Sparrow Search Algorithm [1,2] or Multi-Task Learning models [3,4]. Publishing these works in a single venue demonstrates to engineers which AI tools have reached the maturity required for laboratory implementation.

The analysis of this collection supports the thesis that integrating AI with materials science is not merely an incremental improvement, but a fundamental paradigm shift. The traditional approach, based on empirical testing of thousands of samples, is being replaced by intentional, digital property design. The application of AI represents a breakthrough in structural design and parameter optimization. Based on the presented works, several dominant research directions can be identified.

**Implementation of Machine Learning (ML) and Artificial Intelligence (AI)**. Artificial Intelligence is employed to optimize manufacturing processes, including the fabrication of functionally graded materials (FGMs) [5] and rotary friction welding [6]. Publication [5] highlights the role of AI in managing the complexity of materials with variable property gradients, which is a critical factor in additive manufacturing (3D printing). ML models such as artificial neural networks, Support Vector Machines (SVMs), and Multi-Task Learning (MTL) algorithms are utilized to predict mechanical properties [3,7,8] and material durability under extreme conditions, such as the frost resistance of concrete [1,9].

The integration of ML facilitates the precise customization of the elastic modulus in lattice materials [7]. Furthermore, the application of Multi-Task Learning in the design of concrete-filled double steel tubes (CFDST) demonstrates AI’s capability to identify nonlinear correlations between structural parameters that traditional design codes cannot directly determine [3,10]. Finally, genetic algorithms (GAs) are implemented to navigate complex design spaces to achieve materials with optimized configurations and target densities [7,11,12].

**Utilization of Digital Twins.** Current research underscores the increasing significance of AI-augmented Digital Twin systems for real-time process parameter optimization [6,13,14,15] and advanced structural health assessment of storage tanks [16]. Remote equipment monitoring can be facilitated through Internet of Things (IoT) [17,18] frameworks. By integrating these applications with AI-driven algorithms, predictive maintenance strategies can be implemented to extend asset service life and minimize operational downtime. Specifically, IoT sensors enable the real-time acquisition and transmission of critical parameters, such as corrosion rates and structural strain, which are essential for effective storage tank diagnostics. The Digital Twin concept serves as a cornerstone for faultless production and reliable operation. Industry 4.0 frameworks rely on these digital reflections of physical processes to facilitate continuous monitoring and instantaneous optimization. By integrating artificial neural networks (ANNs) with the Finite Element Method (FEM), it is possible to develop a Digital Twin for the rotary friction welding process capable of optimizing parameters in less than 0.1 s [6]. Such solutions enable highly effective adaptive process control. Similarly, numerical simulations of the lost-foam casting process are utilized to eliminate structural defects in critical components for electric vehicles [19]. Modern maintenance systems, particularly for large-scale storage infrastructure, are evolving toward autonomous ecosystems. The integration of robotics (drones), 3D scanning, and AI-driven processing of non-destructive testing (NDT) data enables the creation of digitized equipment models. These systems facilitate predictive maintenance by autonomously estimating the remaining useful life of the assets [16].

**Advanced Numerical Simulations (FEM/FEA).** The Finite Element Method (FEM) remains the cornerstone for analyzing stress distributions, heat transfer, and structural deformations. It is extensively employed in pavement engineering and design [20,21,22,23,24,25], the thermal insulation analysis of building envelopes [26,27], and the optimization of casting processes for the automotive industry [19]. Specifically, these numerical frameworks allow for the evaluation of complex material behaviors, such as temperature-dependent moduli in asphalt mixtures and heat flow through multi-layered masonry walls, ensuring structural integrity and energy efficiency. In the context of aerospace engineering, publication [28] demonstrates the efficacy of high-fidelity simulations in optimizing stiffened grid panels [29,30], where numerical modeling is essential for balancing weight reduction with structural integrity.

**Modeling of Mechanical Properties and Durability.** A significant number of studies focus on damage prediction and structural integrity. Numerical models facilitate a deeper understanding of crack initiation mechanisms in compacted graphite iron (CGI) [31,32], as well as the effects of non-uniform longitudinal crack width distribution on the degradation of the rebar–concrete bond in corrosion-affected structures [33,34]. Furthermore, in the aerospace sector, the Hartman–Schijve crack growth equations are employed to support the durability analysis and airworthiness certification of repairs performed using the cold spray deposition method [35,36,37]. These approaches allow for a more precise estimation of service life by accounting for microstructural variability and environmental degradation.

**Sustainability and Environmental Engineering.** Research in this field encompasses the utilization of industrial waste for the production of eco-friendly self-compacting concrete [38,39] and the evaluation of storage conditions’ impact on the thermal properties of insulation materials, a factor directly influencing energy efficiency and greenhouse gas emission reduction [11,27]. Studies on thermal insulation (including aerogels, resol boards, and expanded polystyrene) combine laboratory measurements of the thermal conductivity coefficient l with numerical heat flow simulations. This integrated approach helps mitigate design errors that lead to interstitial condensation and excessive energy loss [26,27,40,41]. A significant contribution of this Special Issue is the application of AI and statistical tools to address environmental challenges and assess material durability in changing climatic conditions.

− *Statistically Assisted Recycling*. The application of experimental–statistical modeling [42] enabled the development of self-compacting concrete incorporating brick powder and recycled sand [38]. These models allow for the prediction of the mixture’s workability and compressive strength, significantly reducing the necessity for extensive laboratory trial batches.
− *Thermal Retrofitting and Building Physics*. Comprehensive analyses of insulation materials, ranging from traditional mineral wool to advanced aerogel blankets [26], are supported by TRISCO and WUFI simulation software. This research demonstrates that even minor storage errors—resulting in a degradation of thermal parameters by up to 19%—can be accurately modeled. Such precision enables enhanced energy management throughout the building’s life cycle [27].

**Durability Under Extreme Conditions.** Artificial Intelligence demonstrates superior performance in scenarios where classical mathematical models fail due to the excessive complexity of environmental variables.

− *Frost Damage Prediction*. The implementation of the Sparrow Search Algorithm-optimized Extreme Learning Machine (SSA-ELM) to predict concrete durability in permafrost regions [1] represents a significant advancement in cold-region civil engineering. This model exhibits higher predictive accuracy compared to standard Long Short-Term Memory (LSTM) networks or Support Vector Machines (SVMs).
− *Soil and Pavement Dynamics*. Research in this area includes the analysis of temperature-dependent viscosity and elastic moduli of asphalt binders [20,43], the load-transfer efficiency of dowel bars in concrete pavements [22,44], and the 3D modeling of permeable pavements [21]. A novel approach based on Network Analysis facilitates a comprehensive understanding of how seasonal moisture fluctuations impact the geotechnical parameters of subgrade soils [45,46]. This insight is critical for designing asphalt pavements with enhanced resistance to reflective and fatigue cracking, particularly when subjected to variable climatic loading [20,21,47].

**Interdisciplinary Applications.** The research spectrum presented is remarkably broad, spanning from medical radiotherapy [48] and aerospace engineering (stiffened panels, wind tunnels) [28,49] to geotechnics and the impact of seasonality on soil properties in road construction [45,50,51].

**Digital tools**. Modern materials engineering relies on the integration of Artificial Intelligence (ML, SSA-ELM, MTL) with advanced numerical simulations (FEM, Monte Carlo [52,53]), enabling the precise design of structural properties and the prediction of durability under extreme conditions [1,3,7,54,55]. Table 1 summarizes the application of digital tools in selected publications.

A pivotal trend is the implementation of Digital Twin systems which, powered by genetic algorithms and neural networks, facilitate real-time manufacturing process optimization and infrastructure diagnostics [6,13,14,15,16]. These tools drastically reduce Research and Development (R&D) cycles, minimize technological defects [19,28], and promote sustainable development through the precise modeling of energy efficiency and material recycling [27,38].

**Efficiency of Research Methodologies.** The efficiency of the research methodologies presented in the analyzed publications is based on complementarity: traditional numerical simulations (FEM, Monte Carlo) provide high physical fidelity and deep insight into microstructures [31,48]; however, they are time-consuming and computationally intensive. In contrast, Machine Learning (ML/AI) algorithms, once trained, offer near-instantaneous property predictions with an average error rate of less than 10%. This enables the rapid screening of thousands of design variants [3,7]. A comparison of the efficiency of these research methods is summarized in Table 2. The highest efficiency is achieved by hybrid systems and Digital Twins. By integrating the computational speed of AI with the accuracy of numerical models, these systems enable real-time optimization of technological processes (with response times below 0.1 s), a feat unattainable by classical experimental or purely numerical methods [1,6,16].

**Synergy Between AI and Materials Engineering**. The synergy between Artificial Intelligence (AI) and materials engineering is manifested in the transition from traditional “trial-and-error” methodologies to intelligent inverse design. In this approach, algorithms precisely determine structural parameters based on predefined target properties [3,7]. The integration of Machine Learning with physical models facilitates the development of Digital Twin systems capable of adjusting manufacturing processes in milliseconds and detecting anomalies invisible to conventional inspection methods [5,6]. This interaction is summarized in Table 3.

This constructive interaction not only drastically accelerates the discovery of novel materials but also revolutionizes infrastructure diagnostics by enabling a shift from reactive repairs to predictive life-cycle management [1,16].

**The Future of AI-Assisted Engineering**. The synthesis of these twenty research papers leads to the conclusion that Artificial Intelligence does not replace the engineer but drastically extends their analytical and design capabilities. The convergence of AI and materials science has transitioned from a theoretical concept to a practical engineering instrument. Contemporary engineering is shifting away from traditional trial-and-error methodologies toward intelligent inverse design. The studies included in this Special Issue demonstrate that integrating experimental data with AI models facilitates the following:

− *Enhanced Efficiency*. AI enables navigation through vast design spaces where the number of chemical compositions or geometric parameters exceeds human cognitive limits. Processes such as casting [19,56,57,58] and pavement engineering [21] gain a new dimension of quality through high-precision predictive modeling.
− *Cost Reduction*. The integration of Finite Element Method (FEM) simulations with Machine Learning (ML), as demonstrated in [7,19], allows for virtual prototyping. This drastically reduces material and energy consumption during the Research and Development (R&D) phase.
− *Improved Safety and Durability*. Utilizing advanced fracture models [31,59] and bond degradation analysis [33,60] enables the design of safer infrastructure supported by Digital Twin systems. Advanced analytics of cracking and corrosion [31,33,35,61] facilitate the construction of more resilient machinery and structures.
− *Environmental Sustainability*. AI-driven optimization of building insulation [27,62] and the implementation of recycled materials [38,63] directly support sustainable development goals.

This Special Issue serves as evidence that we have reached a pivotal turning point where materials engineering is evolving into a digital discipline. This transformation enables the development of technologies that were considered impossible to optimize only a decade ago. The presented research provides a robust knowledge platform that stimulates further innovation in materials science, addressing the challenges of modern industry and environmental protection.

**Perspective on Interdisciplinary Collaboration.** This Special Issue serves as a unique platform bridging the gap between Artificial Intelligence theorists and materials science practitioners. For AI researchers, it provides a rich repository of complex physical problems and unique datasets essential for validating cutting-edge algorithms. Conversely, for materials scientists, these publications offer an inspiring roadmap for transitioning from traditional, empirical methodologies to the active integration of digital tools within standard laboratory workflows. This constructive interaction facilitates the dismantling of disciplinary silos and promotes the advancement of Explainable Artificial Intelligence (XAI). By aligning mathematical model precision with physical interpretability, XAI ensures that data-driven insights remain consistent with fundamental material phenomena.

## Figures and Tables

**Table 1:** Application of digital tools in selected publications.

Field	Technologies/Methods	Publications
Machine Learning/AI	Neural networks (ANN), MTL, SSA-ELM, SHAP interpretability	[1,3,5,6,7]
Numerical Methods	FEM, FEA, Monte Carlo method, ProCAST software	[16,19,21,22,26,27,31,38,48]
Process Optimization	Digital Twins, genetic algorithms (GAs)	[6,7,16]
Mathematical Modeling	Regression models, orthogonal functions, Hartman–Schijve equations	[20,35,49]

**Table 2:** Comparison of research methodology efficiency.

Method	Advantages According to Analyzed Studies	Exemplary Application
Monte Carlo	High precision in modeling particle energy distribution.	FLASH radiotherapy [48]
Machine Learning (ML)	Ability to map nonlinear relationships and rapid prediction.	CFDST design [3], Frost resistance [1]
FEM + AI Hybrid	Combines physical interpretability with computational speed.	Digital Twins in welding [6]
Statistical Modeling	Multi-objective optimization of waste-based material compositions.	Brick-dust-modified concrete [38]

**Table 3:** Synergy between AI methods and materials science and engineering.

Publication	Material Challenge	Applied AI Tool/Model	Key Result/Innovation
[1]	Concrete frost action	SSA-ELM (Sparrow Search Algorithm)	State-of-the-art precision in predicting frost damage within permafrost.
[3]	CFDST structures	Multi-Task Learning (Lasso, VSTG, MLS-SVR)	Identification of latent correlations between geometric parameters and strength.
[5]	FGM fabrication	Machine Learning (ML)	Optimization of 3D printing parameters and real-time defect detection.
[6]	Ti/Al dissimilar welding	Digital Twin + ANN + Genetic Algorithm	Process optimization in <0.1 s; elimination of weld defects.
[7]	Lattice customization	Two-Tier ML (Polynomial Regression)	25% improvement in mechanical performance at constant density.
